# tRNA binding to Kti12 is crucial for wobble uridine modification by Elongator

**DOI:** 10.1093/nar/gkaf296

**Published:** 2025-04-14

**Authors:** David Scherf, Alexander Hammermeister, Pauline Böhnert, Alicia Burkard, Mark Helm, Sebastian Glatt, Raffael Schaffrath

**Affiliations:** Institute of Biology, Division of Microbiology, University of Kassel, D-34132 Kassel, Germany; Institute of Biology, Division of Microbiology, University of Kassel, D-34132 Kassel, Germany; Małopolska Centre of Biotechnology, Jagiellonian University, 30387 Krakow, Poland; Institute of Biology, Division of Microbiology, University of Kassel, D-34132 Kassel, Germany; Institute of Pharmaceutical and Biomedical Sciences, Johannes Gutenberg University of Mainz, D-55128 Mainz, Germany; Institute of Pharmaceutical and Biomedical Sciences, Johannes Gutenberg University of Mainz, D-55128 Mainz, Germany; Małopolska Centre of Biotechnology, Jagiellonian University, 30387 Krakow, Poland; Department for Biological Sciences and Pathobiology, University of Veterinary Medicine Vienna, Vienna, Austria; Institute of Biology, Division of Microbiology, University of Kassel, D-34132 Kassel, Germany

## Abstract

In yeast, tRNA modifications that are introduced by the Elongator complex are recognized by zymocin, a fungal tRNase killer toxin that cleaves the anticodon. Based on zymocin resistance conferred by mutations in *KTI12*, a gene coding for an Elongator interactor, we further examined the yet vaguely defined cellular role of Kti12. Guided by structural similarities between Kti12 and PSTK, a tRNA kinase involved in selenocysteine synthesis, we identified conserved basic residues in the C-terminus of Kti12, which upon site-directed mutagenesis caused progressive loss of tRNA binding *in vitro*. The inability of Kti12 to bind tRNA led to similar phenotypes caused by Elongator inactivation *in vivo*. Consistently, tRNA binding deficient *kti12* mutants drastically suppressed Elongator dependent tRNA anticodon modifications and reduced the capacity of Kti12 to interact with Elongator. We further could distinguish Elongator unbound pools of Kti12 in a tRNA dependent manner from bound ones. In summary, the C-terminal domain of Kti12 is crucial for tRNA binding and Kti12 recruitment to Elongator, which are both requirements for Elongator function suggesting Kti12 is a tRNA carrier that interacts with Elongator for modification of the tRNA anticodon.

## Graphical Abstract

**Graphical Abstract:**
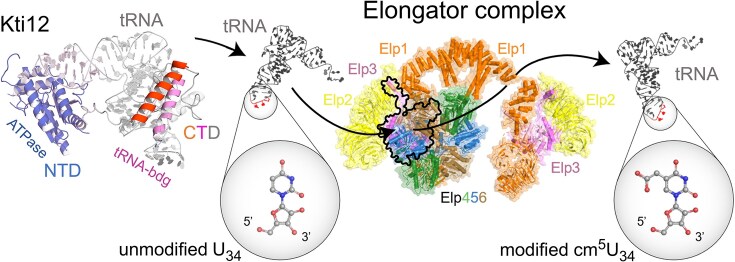

## Introduction

Recent cryo-EM structures have revealed that the architecture of the eukaryotic Elongator complex is highly conserved among species [1–4]. Elongator is composed of two modules, namely Elp123 and Elp456, and their function in tRNA modification can be exchanged between lower and higher eukaryotes [5–8]. In detail, the Elongator complex decorates uridine bases in the anticodon wobble position (U_34_) of several tRNAs with 5-carboxy-methyl (cm^5^U_34_) groups [5,9]. Subsequently, these can be derivatized in concert with other tRNA modifiers, to complex U_34_ modification types such as 5-carbamoylmethyl (ncm^5^U_34_), 5-methoxycarbonylmethyl (mcm^5^U_34_), or 5-methoxycarbonylmethyl-2-thio (mcm^5^s^2^U_34_) [9–11].

Physiologically, U_34_ modifications tune anticodon-codon interactions during mRNA translation and protect against codon-specific ribosome pausing [12–14]. Hence, loss of U_34_ modifications compromises translation fidelity and protein homeostasis, causing pleiotropic stress-induced growth phenotypes [15–17]. In yeast, these can at least in part be rescued by overexpression of elongation factor 1A or Elongator substrate tRNAs, implying that enhanced tRNA delivery to the ribosome can compensate for inefficient translation rates associated with U_34_ modification loss [13, 18, 19]. Inappropriately altered U_34_ modification levels are found in animal models for human diseases and in patients suffering from intellectual disability, neuropathies and certain cancers [20–25]. This proves clinical importance of understanding the complete U_34_ modification pathway and suggests that only constant Elongator activity ensures proper protein synthesis rates, particularly in neuronal cells [11, 25–27].

However, there is also evidence showing that U_34_ modifications can change during the cell cycle and in response to varying environmental conditions [28–32]. It is important to mention that several accessory proteins with roles in Elongator regulation have been identified in yeast [17, 27]. They include casein kinase 1 (Hrr25/Kti14), type 2A phosphatase (Sit4) and Kti12, which is related to PSTK (*O*-phosphoseryl-tRNA^Sec^-kinase), a tRNA kinase needed for selenocysteine synthesis [33–37]. They all interact with Elongator and affect the phosphorylation state of its Elp1 subunit, suggesting that its activity is under phospho-regulation in yeast [35, 38]. In support of this notion, functionally relevant phosphorylation sites in Elp1 map near a tRNA binding domain that is essential for Elongator to modify U_34_. Of note, Hrr25 kinase recruitment to Elongator depends on the presence of Kti12 [38, 39], suggesting that a complex interplay of the regulatory factors exists. Sequence homology and partial crystal structures show that Kti12 contains N- and C-terminal domains (NTD/CTD). These two domains, like in PSTK, are connected by a linker region and carry out ATPase and tRNA binding activities, respectively [36, 40, 41]. Markedly, the ATPase activity of Kti12 can be stimulated in the presence of tRNAs *in vitro* and is crucial for Elongator dependent U_34_ modification *in vivo* [41, 42]. How the NTD and CTD are functionally and mechanistically coupled in Kti12 to support U_34_ modification by Elongator, remains unclear.

Here we present a comprehensive study to clarify the mechanism of tRNA binding by Kti12 and identify individual residues in its CTD responsible for tRNA binding using site-specific mutagenesis. In addition, we examine the consequences that alterations of these residues have on Kti12 interaction with Elongator. Finally, we describe the significance of tRNA binding by Kti12 for the U_34_ modification activity of the Elongator complex *in vivo*. Together with Elongator bound and unbound fractions of Kti12 that can be distinguished in a tRNA dependent fashion, our data confirm that Kti12, through direct tRNA binding, enables Elongator to modify tRNA anticodons *in vivo*.

## Materials and methods

### Yeast genetic manipulation and phenotypic characterization

*Saccharomyces cerevisiae* strains (Supplementary Table S1) were generated as previously described [41]. Briefly, genes of interest were deleted using primers (Supplementary Table S2) to amplify a *KlURA3* marker cassette by polymerase chain reaction (PCR) with homology to the targeted loci including *KTI12*. Similarly, epitope-tags were introduced [43] at the C-termini of gene products of interest via PCR and primers (Supplementary Table S2) followed by yeast genome insertion. Site-directed mutagenesis used PCR and primers (Supplementary Table S2) as described [44] with a template plasmid carrying *KTI12-HA::KlTRP1*; the resulting PCR products were reinserted into the *kti12*Δ strain. Yeast transformants were selected for tryptophan prototrophy and 5-fluoroorotic acid resistance, confirmed by PCR and DNA sequencing. All yeast strains were generated in UMY2893 [5] containing the Elongator-dependent *ochre* (UAA) tRNA suppressor (*SUP4*) and suppressible *ade2-1^ochre^* and *can1-100^ochre^* reporter genes. To analyze Elongator function on the basis of *SUP4* assays, yeasts were grown in synthetic complete media containing 2% glucose (w/v), 0.7% yeast nitrogen base (w/v), supplemented with or without adenine and canavanine. Functional Elongator and mcm^5^U_34_ modification of *SUP4* enable stop codon read-through of *ade2-1^ochre^* and *can1-100^ochre^*, which can be assessed by adenine prototrophy or canavanine sensitivity [5]. Galactose induced expression of γ-toxin was achieved by transformation with pLF16 [45] and incubation on media lacking leucine at 30°C for at least 2 days. Exogenous zymocin was prepared by growing the *Kluyveromyces lactis* killer strain AWJ137 (Supplementary Table S1) at 30°C for 2 day [17] and ultrafiltration of culture supernatant using a 50 kDa molecular weight cut-off (MWCO) filter [46]. Zymocin was plated on rich media (2% glucose (w/v), 2% tryptone (w/v), 1% yeast extract (w/v), 2% agar-agar (w/v)) in different concentrations. Yeast cells were spotted as ten-fold serial dilutions and grown in the presence (or absence) of zymocin for 2 days at 30°C.

### γ-toxin tRNA cleavage and LC-MS/MS quantification of tRNA modifications

To estimate the amount of mcm^5^s^2^U_34_-modified tRNA, bulk tRNA was purified and cleaved by γ-toxin *in vitro* and analyzed as previously described [3, 4]. Nucleoside levels were analyzed via liquid chromatography-tandem mass spectrometry (LC-MS/MS) as reported previously [47] with the sample amount being adjusted to 1 μg of digested tRNA spiked with 100 ng of internal standards (digested ^13^C-labeled tRNA from *S. cerevisiae*). For absolute quantification of biological duplicates in technical triplicates, internal and external calibration with synthetic standards was applied as detailed in [48]. Of note, the internal standard did not contain s^2^U, which is why s^2^U calculations were performed with external calibration only. Finally, the total levels of modified nucleosides were normalized to the amount of uridines and related to the corresponding reference sample (set to 1).

### Yeast extract preparation

Yeast cells were grown to mid-logarithmic phase, centrifuged for 2 min (4°C) and washed. Centrifugation was repeated and supernatants discarded. Next, cells were resolubilized in B60 buffer (50 mM HEPES-KOH pH 7.3 at 4°C, 60 mM KOAc, 5mM Mg(OAc)_2_, 0.1% (v/v) Triton^TM^ X-100, 10% (v/v) glycerol, 1 mM NaF, 20 mM ß-glycerolphosphate, 1 mM DTT) and 300 μl per 50 OD_600_ units of cOmplete^TM^ protease inhibitor cocktail (Roche) were added. Cells were lysed by adding 300 μl glass beads (0.5 mm diameter) under vigorous shaking in the Mini Bead-beater for 60 s [49] and chilled on ice for 5 min. Shaking was repeated six times, after which a centrifugation at 4°C 15 000 rpm for 10 min was carried out. The lysate was transferred to a new reaction tube and another centrifugation at 4°C 15 000 rpm for 30 min was performed to clear the lysis. The cleared supernatant was pooled, protein concentration determined by Bradford [50] and frozen at –20°C for further analysis.

### Co-immune precipitation (Co-IP) experiments

For co-immune precipitation (Co-IP), 100 μg antibodies of choice were amide coupled to 20 mg magnetic Dynabeads^TM^ M270 Epoxy (Thermo Scientific) according to the manufacturer's instruction resulting in a final concentration of 5 μg antibody/mg Dynabeads^TM^. Typically, 8 mg of total yeast protein were used for the Co-IP experiment. Protein extracts were adjusted in B60 buffer and as a loading control, 1/10 was denatured with Laemmli buffer (60 mM Tris HCl pH 6.8 at 4°C, 1% (w/v) sodium dodecyl sulfate (SDS), 5% (v/v) glycerol, 2.5% (v/v) ß-mercaptoethanol, 0.001% (v/v) bromophenol blue) at 99°C. The remaining protein isolate was incubated with 20 μl antibody coupled Dynabeads or anti-FLAG® M2 magnetic beads (Sigma-Aldrich) at 4°C overnight. After incubation, beads were retained by a magnet and washed thrice with 500 μl B60 buffer at 4°C. Protein elution used incubation with 64 μl IP Elution buffer (50 mM Tris HCl pH 8.0 at rt, 0.2% w/v SDS, 0.1% (v/v) Tween) at 50°C for 10 min and eluates were denatured with Laemmli buffer as above. Input and bead-precipitated protein were subjected to SDS-polyacrylamide gelelectrophoresis (PAGE) and Western blot analyses.

### Western blotting procedures

Protein solutions were incubated with denaturing Laemmli buffer at 99°C for 10 min. The mixture was then loaded onto a discontinuous SDS-PAGE with a stacking gel (6% (w/v) acrylamide (acrylamide/bis-acrylamide 37.5:1), 1x stacking buffer (125 mM Tris HCl pH 6.8 at rt, 0.1% (w/v) SDS), 0.003% (w/v) bromophenol blue, 0.1% (w/v) APS, 0.05% (v/v) TEMED) and a separating gel (12% (w/v) acrylamide (acrylamide/bis-acrylamide 37.5:1), 1x separation buffer (375 mM Tris HCl pH 8.8 at rt, 0.1% (w/v) SDS, 0.1% (w/v) APS, 0.05% (v/v) TEMED). The gel was run at 200 V for ∼60 min in SDS running buffer (25 mM Tris, 192 mM glycine, 0.1% (w/v) SDS). Proteins were blotted from the gel to a 0.45 μM pore sized polyvinylidene fluoride (PVDF, Merck Millipore) membrane using a Trans-Blot® Turbo^TM^ apparatus (Bio-Rad) and Bjerrum Schafer-Nielsen buffer (48 mM Tris, 39 mM glycine, 0.1% (w/v) SDS, 20% (v/v) EtOH) [51]. Antibodies immune detection of epitope-tagged proteins of interest or pyruvate kinase (Cdc19) were anti-c-Myc (9E10; Santa Cruz), anti-HA (DLN-012263 Dianova), anti-His (PA1-983B, Thermo Fisher) and anti-Cdc19 (donated by Dr J. Thorner).

### Recombinant protein purification and GST/strep-pulldown

Recombinant Kti12 and Elp1 domains were purified as described [41]. Purification of the γ-toxin-GST fusion used agarose matrix bound to glutathione (Protino® GST / 4B, Macherey-Nagel). Bacterial lysate was produced as described [41] and diluted 1:10 in GST wash buffer (50 mM Tris HCl pH 7.5 at rt, 300 mM NaCl, 2 mM DTT). To remove precipitate, the solution was spun down at 4°C and 4000 rpm for 10 min. The supernatant was loaded onto the Protino® GST column and circulated for at least 2 h. Next, the column was washed with 8 column volumes (cv) of GST wash buffer. The Protino® GST column was eluted with 10 cv GST elution buffer (50 mM Tris HCl pH 7.5 at rt, 300 mM NaCl, 2 mM DTT, 20 mM glutathione) and protein was concentrated using a 30 kDa MWCO. γ-toxin was further purified using a Sepharose 75 (HiLoad® 16/600 Superdex® 75 pg) column and eluted with SEC buffer (50 mM Tris HCl pH 7.5 at rt, 150 mM NaCl, 2 mM DTT). Fractions with the desired protein were pooled. GST/Strep-tagged proteins were incubated with Protino glutathione agarose 4B (Macherey Nagel)/ Strep-Tactin^TM^XT4Flow^TM^ (IBA-Lifesciences) beads overnight at 4°C, washed three times with pulldown buffer (50 mM Tris HCl pH 7.5 at rt, 150 mM NaCl, 2 mM DTT, 0.1% Triton X-100) and precipitated from the beads by incubation in 1x Laemmli buffer at 99°C for 5 min. Precipitates were controlled by SDS-PAGE and Coomassie staining.

### Electrophoretic mobility shift assays

tRNA-protein interaction was analyzed by the change in electrophoretic mobility of tRNA when bound to a protein. Therefore, recombinantly purified protein was incubated with 80 nM bulk tRNA purified from yeast in 20 mM Tris-HCl pH 7.5 at 4°C, 150 mM NaCl, 2 mM DTT, 1 MgCl_2_ for 30 min at rt. Samples were split in half and controlled for protein loading via SDS-PAGE. The other half was loaded onto native PAGE together with 10% sucrose (45 mM Tris-HCl pH 7.5 at 4°C, 45 mM boric acid, 10% sucrose, 5% acrylamide (19:1), 0.1% APS, 0.1% TEMED). Native PAGE was pre-run in 45 mM Tris-HCl pH 7.5 at 4°C, 45 mM boric acid at 60 V for 60 min at 4°C and samples were resolved at 60 V for 90 min at 4°C. The electrophoretic mobility shift assay (EMSA) gel was stained in SYBR^TM^-Gold and tRNA was visualized via an LED transilluminator (FastGene).

### UV crosslinking of proteins to nucleic acids

UV crosslinking of immunoprecipitated proteins was as described [52] and performed after a sequential IP on two Kti12 containing fractions. Yeast cells co-expressing Elp1-c-Myc and Kti12-HA were grown, harvested and washed as described above. Cells were lysed in ice cold IPP100 buffer (10 mM Tris-HCl pH 8.0 at RT, 100 mM NaCl, 2 mM MgCl_2_, 0.1% (v/v) Nonidet ^TM^*P*-40, 1 mM DTT) supplemented with cOmplete^TM^ protease inhibitor cocktail. For the first IP, 10 mg protein extract was incubated with anti-c-Myc coupled Dynabeads for 30 min at room temperature, to generate the Elp1-bound fraction. The bound fraction was collected using a magnetic rack and the remaining Elp1 immunodepleted protein extract was removed into a new reaction tube, to precipitate Elp1-free Kti12 using anti-HA coupled Dynabeads, for 30 min at room temperature. Both fractions were washed four times with IPP300 buffer (same as IPP100 buffer, but 300 mM NaCl). Elp1-bound and Elp1-free fractions were resuspended in a small volume of IPP100 buffer and split in two (control and UV irradiated). The control fraction was kept on ice and the second fraction was irradiated with UV light on ice (254 nm, 3.2 J/cm2 at 10 cm distance). Both samples were washed, resuspended in IPP100 buffer with Laemmli buffer, incubated at 99°C for 10 min and subjected to Western blot analysis.

### IP based assay for Kti12 to association Elp1

The impact of tRNA and/or adenosine nucleotides (AxP) on Kti12 interaction with Elp1 used immune-precipitated Elp1-c-Myc from *kti12*Δ cells and recombinant Kti12 for association studies *in vitro*. Kti12 was pre-incubated on ice, either mock or with AxP and in absence or presence of bulk yeast tRNA. A 25 μl preassociation reaction consists of ice cold IPP100 buffer containing 5 μM Kti12 together with 2 mM AxP (Jena Bioscience) and ± 3 μM tRNA. The sample was diluted to 250 μl with 1/9^th^ volume bound of Elp1-c-Myc so that each reaction contained an amount equivalent to an IP from 1 mg protein extract. Untreated Elp1-c-Myc served as input control without Kti12. The association reactions were incubated for 60 min at 4°C under constant rotation. Elp1 and associated Kti12 were collected using a magnetic rack. The supernatant was discarded and samples washed twice in ice cold IPP100 buffer. The samples were resuspended in IPP100 and Laemmli buffer, incubated at 99°C for 10 min and subjected to Western blot analysis.

### Protein modeling and software

Kti12 was modeled using SWISS-MODEL [53] and aligned to the MjPSTK (3ADB) structure and CtKti12 (6QP0) using PyMOL. Sequences of Kti12 like proteins were aligned using MAFFT [54] and the FFT-NS-i algorithm.

## Results and discussion

### Identification of conserved and functional relevant residues in the CTD of Kti12

**Figure 1.**
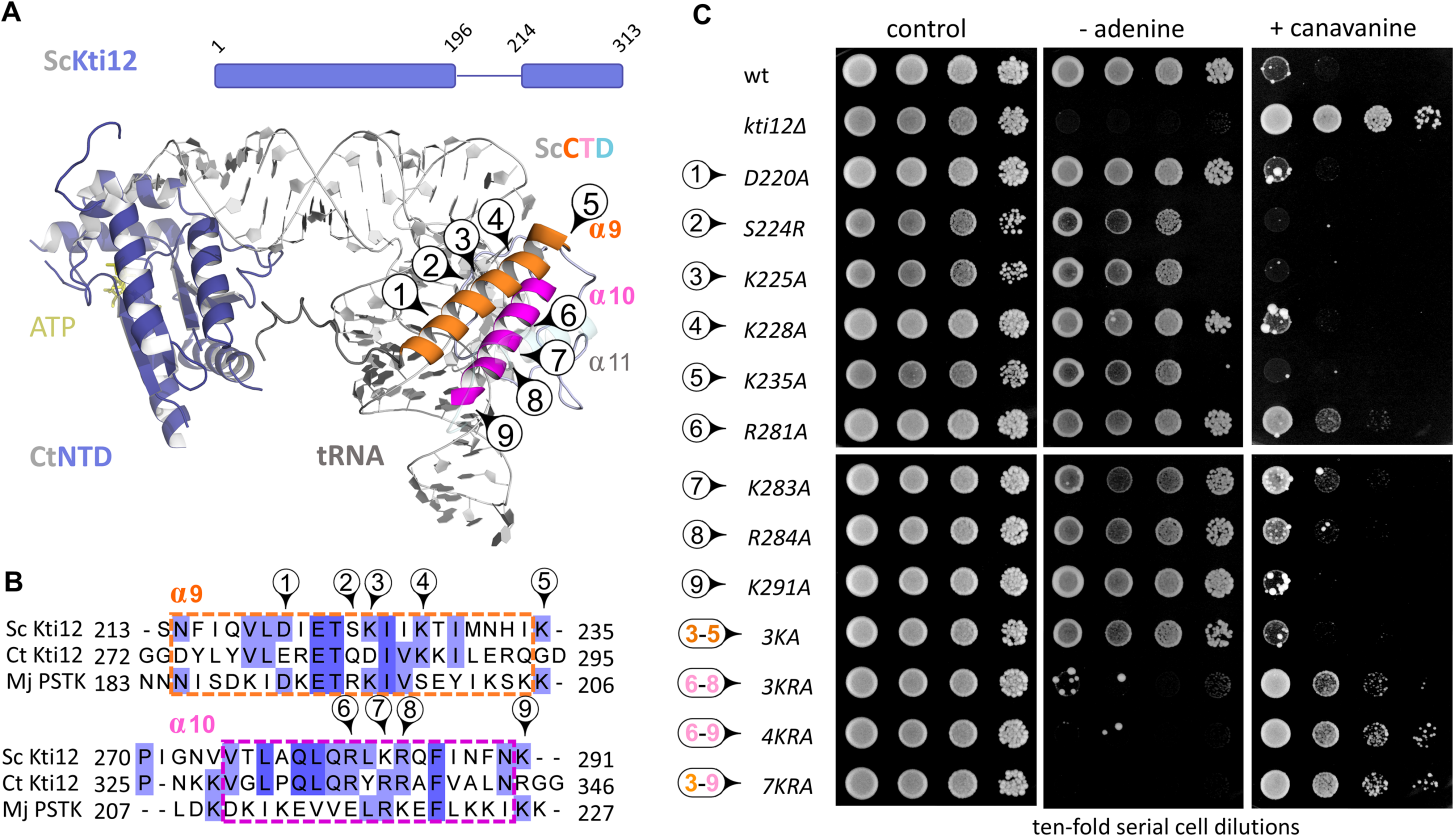
Domain conservation with PSTK identifies putative tRNA binding residues in the CTD of Kti12. **(A)** Structural Kti12 model showing conserved α-helices (α-9 orange, α-10 magenta) in the CTD with candidate tRNA binding roles. **(B)** Alignment and selection of conserved residues in α-9/α-10 (see A) of ScKti12 for substitution mutagenesis. **(C)***SUP4ochre*stop-codon read-through analysis of *ade2-1*^*ochre*^ and *can1-100*^*ochre*^ reporter genes involved *kti12* substitution mutants, wild-type (wt) or *kti12*Δ controls in order to assess Kti12 and Elongator function *in vivo* based on adenine prototrophy and canavanine sensitivity (control: synthetic complete medium; for details see the “Materials and methods” section.).

PSTK from *Methanocaldococcus jannaschii* (MjPSTK) binds ATP via its NTD, which is connected to the CTD via a flexible linker [40]. Previously, comparative analysis had suggested that the CTDs in both, PSTK and Kti12, are involved in tRNA binding [40, 41]. Guided by the resolved structures of full-length MjPSTK and the NTD of Kti12 from *Chaetomium thermophilum* (CtKti12), we generated a structural model of Kti12 from *S. cerevisiae* (ScKti12) in its tRNA bound form (Fig. 1A and Supplementary Fig. S1A). We identified several conserved CTD residues in the projected helices α9 (Asp-220, Ser-224, Lys-225, Lys-228) and α10 (Lys-235, Arg-281, Lys-283, and Lys-284, Lys-291), which in MjPSTK are known to be crucial for tRNA binding [40] (Fig. 1B).

These residues were individually substituted by alanine at the genomic *KTI12* locus, except for Ser-224, which was replaced by arginine to imitate the corresponding site in MjPSTK (Fig. 1B). In addition, substitutions of individual residues within helix α9 and/or α10 were also combined in mutants to create multiple replacements (3KA, 3KRA, 4KRA, and 7KRA) (Fig. 1C). Next, we examined the effect of all substitutions generated on Elongator function *in vivo*. In detail, we used the *SUP4* tRNA^Tyr^_UΨA_ system (with the critically modified U_34_ underlined). *SUP4* promotes read-through of *ochre* (UAA) stop codons in *ade2-1^ochre^* and *can1-100^ochre^* reporter genes dependent on U_34_ modifications and therefore, allows to monitor Elongator activity by conferring adenine prototrophy and canavanine sensitivity, respectively, when U_34_ is modified to mcm^5^U_34_ [5, 45]. Our data show a similar level of canavanine resistance between the *kti12*Δ control and the combined CTD substitutions (3KRA, 4KRA, and 7KRA), which point to an Elongator-linked U_34_ modification defect due to Kti12 inactivation (Fig. 1C). The same CTD mutations (i.e. 3KRA, 4KRA, and 7KRA) were found to be as auxotrophic for adenine as the *kti12*Δ control (Fig. 1C), again indicating Elongator loss-of-function and failure of *ade2-1^ochre^* read-through by *SUP4*. Collectively, our data indicate that while individual substitutions in the CTD of Kti12 (D220A, S224R, K225A, K228A, K235A, R281A, K283A, R284A, and R291A) do not elicit Elongator-minus phenotypes, their combination, in particular replacements of basic residues in helix α9 and/or helix α10 (3KRA, 4KRA, and 7KRA), reduces Elongator function *in vivo* (Fig. 1C). We conclude that multiple substitutions in the candidate tRNA binding region of Kti12 trigger phenotypes typically associated with Elongator-loss-of-function mutants (e.g. *elp3*Δ).

**Figure 2.**
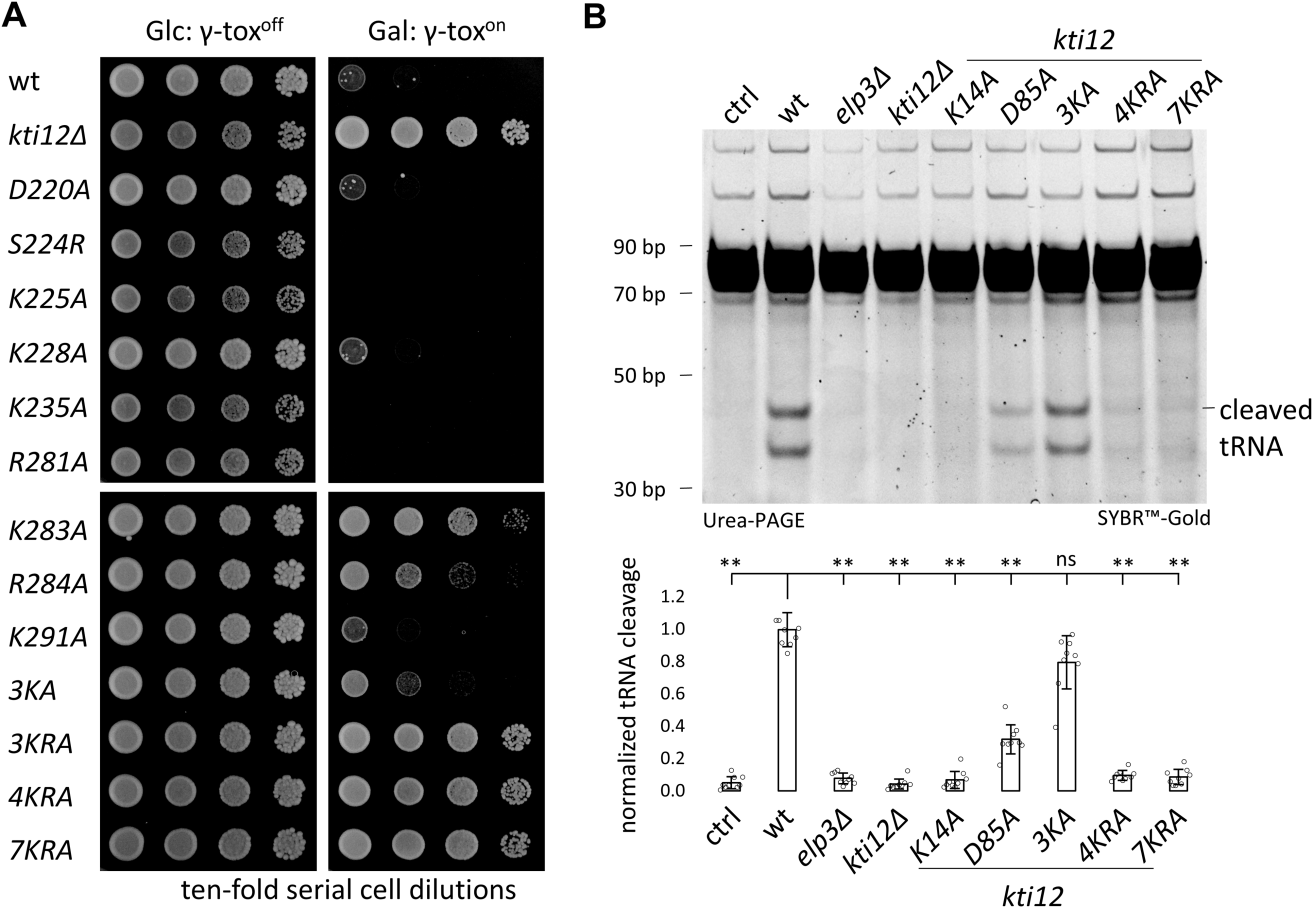
CTD integrity is crucial for Kti12 and Elongator function based on mcm^5^s^2^U_34_ dependent γ-toxin tRNase activity. **(A)** Galactose-dependent expression of the tRNase γ-toxin identifies loss-of-function phenotypes among the indicated *kti12* substitution mutants. Their resistance to growth inhibition is in contrast to the *KTI12* wild-type (wt) with normal Elongator dependent mcm^5^s^2^U_34_ modification capacity. **(B)** Assay for mcm^5^s^2^U_34_ cleavage by γ-toxin *in vitro* (upper panel) and quantification (lower panel) of tRNA cleavage efficiencies. Compared are tRNAs purified from previously characterized N-terminal Kti12-ATP binding pocket mutants (K14A, D85A) and selected CTD substitution mutants (3KA, 4KRA, 7KRA) generated in this report. As a control (ctrl), the wt sample was incubated without γ-toxin. In the lower panel, error bars indicate standard deviation. Statistical significance was tested using a two-tailed t-test (** *P* < 0.01, * *P* < 0.05, ns: not significant).

To further study the consequences of CTD substitutions on Kti12 function and U_34_ modification levels, we employed zymocin, a tRNase killer toxin complex, which requires Elongator dependent mcm^5^s^2^U_34_ modifications for anticodon cleavage [27, 55]. Yeast cells were transformed with a conditional expression plasmid, allowing for galactose dependent production of γ-toxin, the active tRNase subunit of zymocin [5, 17, 56]. Next, the growth of the various strains was compared under repressing and inducing conditions (Fig. 2A). On galactose medium, the behavior of most single CTD substitution mutants (i.e. D220A, S224R, K225A, K228A, K235A, R281A, and R291A) recapitulated *KTI12* wild-type cells, showing strongly inhibited growth by the γ-toxin (Fig. 2A). This result indicates that the U_34_ modification function of Elongator, which is required for tRNase activity of γ-toxin, is hardly altered by individual amino acid changes in the CTD (Fig. 2A). Of note, two single (i.e. K283A and R284A) and one patch mutation (3KA) showed partial phenotypes. However, all other mutants carrying multiple combined substitutions (3KRA, 4KRA, 7KRA) (Fig. 2A) displayed full resistance to γ-toxin. In fact, their response recapitulates the resistance of the *kti12*Δ control, lacking Elongator activity (Fig. 2A). In summary, the observed phenotypes are consistent with loss-of-Elongator activity in the *SUP4* read-through assays (Fig. 1C). To complement the γ-toxin assays, growth inhibition was also assessed using zymocin purified from culture filtrates of the *K. lactis* killer strain. Again, among all mutants tested, the CTD patch substitutions (i.e. 3KRA, 4KRA, 7KRA) conferred zymocin resistance similar to the *kti12*Δ control (Supplementary Fig. S1B).

Furthermore we performed *in vitro* tRNA cleavage assays, which use purified γ-toxin [3, 4, 56, 57] to compare consequences on U_34_ modification between mutants in the CTD (i.e. 4KRA, 7KRA) with previously established mutants in the NTD of Kti12 (i.e. K14A and D85A) that are known to be defective in ATP binding and ATPase activity [41]. Isolated bulk tRNA from an Elongator wild-type strain is efficiently cleaved by purified γ-toxin, which produces cleavage products for mcm^5^s^2^U_34_ modified anticodons (Fig. 2B and Supplementary Fig. S2). tRNA from the D85A strain shows significantly less tRNA cleavage than *KTI12* wild-type cells, while tRNAs from the K14A mutant are fully resistant against tRNA cleavage (Fig. 2A). The combined CTD substitutions (i.e. 4KRA, 7KRA) do not show tRNA cleavage *in vitro* (Fig. 2B), which confirms that Elongator activity is lost when multiple substitution mutations are introduced into the CTD of Kti12 (Fig. 2A). Thus, our data strongly support the option that similar to PSTK [40], the CTD as well as the NTD are both required for full-length Kti12 to function and support the U_34_ modification activity of the Elongator complex *in vivo*.

### Kti12 binds tRNA through basic residues in its CTD

**Figure 3.**
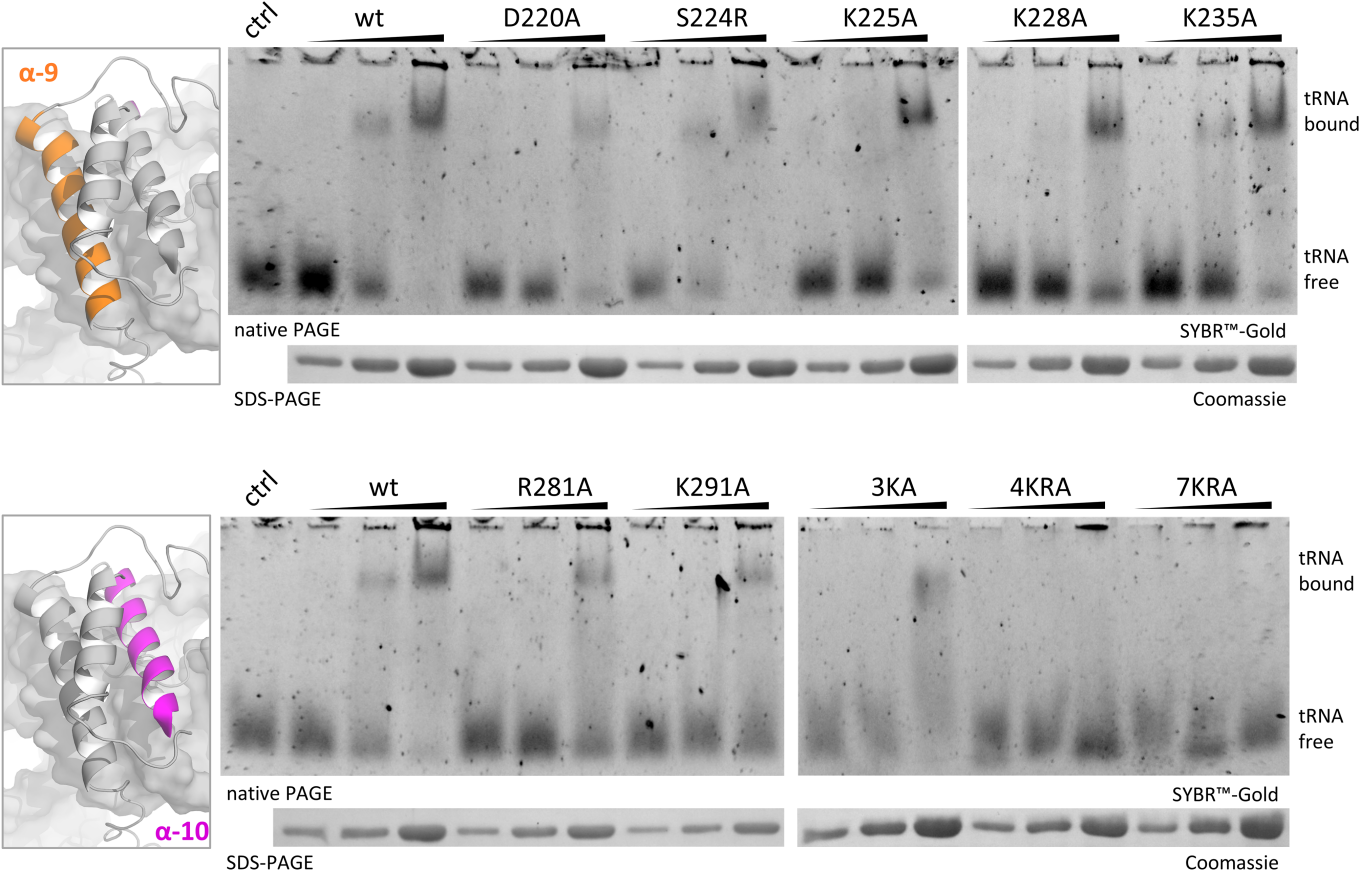
The CTD of Kti12 binds tRNA. The affinity of the indicated CTD mutants to tRNA was examined by EMSA together with the wild-type (wt) protein. Increasing concentrations of recombinant Kti12 (1.5, 3, and 7.5 μM) were incubated with 55 nM wt bulk tRNA and run on a 5% native PAGE. As control (ctrl), a sample without protein was used. tRNA mobility and capture by Kti12 was detected via SYBR Gold staining, whereas protein loading was controlled via SDS-PAGE and Coomassie staining.

To elucidate the mechanistic impact of CTD substitutions on Kti12 function, EMSA were carried out that probe for tRNA binding capacity *in vitro* (Fig. 3). Using mixtures of bulk tRNA isolated from yeast and different concentrations of the Kti12 variants purified from *Escherichia coli*, we observed robust tRNA binding activity with the purified ScKti12 wild-type protein (Fig. 3). This finding reconfirms our previous report [41] demonstrating that Kti12 from *C. thermophilum* binds bulk yeast tRNA. Recombinant mutants with single CTD substitutions in helix α9 or α10 show unaffected (i.e. S224R, K235A) or only slightly decreased (i.e. D220A, K225A, K228A, R281A, and R291A) tRNA binding compared to wild-type Kti12 (Fig. 3). In contrast to these mild effects, the combined CTD substitutions in helices α10 (4KRA) or α9/α10 (7KRA) have a dramatic effect on tRNA binding (Fig. 3). The respective Kti12 mutants (i.e. 4KRA and 7KRA) have lost the ability to bind tRNA *in vitro* (Fig. 3). Thus, basic CTD residues in helices α9 and/or α10 (Fig. 1B), collectively mediate tRNA binding by Kti12. Moreover, their failure to bind tRNA *in vitro* (Fig. 3), might as well explain the Elongator-minus phenotypes shown to be triggered by the very same mutations *in vivo* (Figs 1 and 2). Therefore, we conclude that CTD-dependent binding of Kti12 to tRNA is indeed crucial for the U_34_ modification activity of Elongator *in vivo*.

### LC-MS/MS reveals U_34_ modification defects due to multiple CTD substitutions

**Figure 4.**
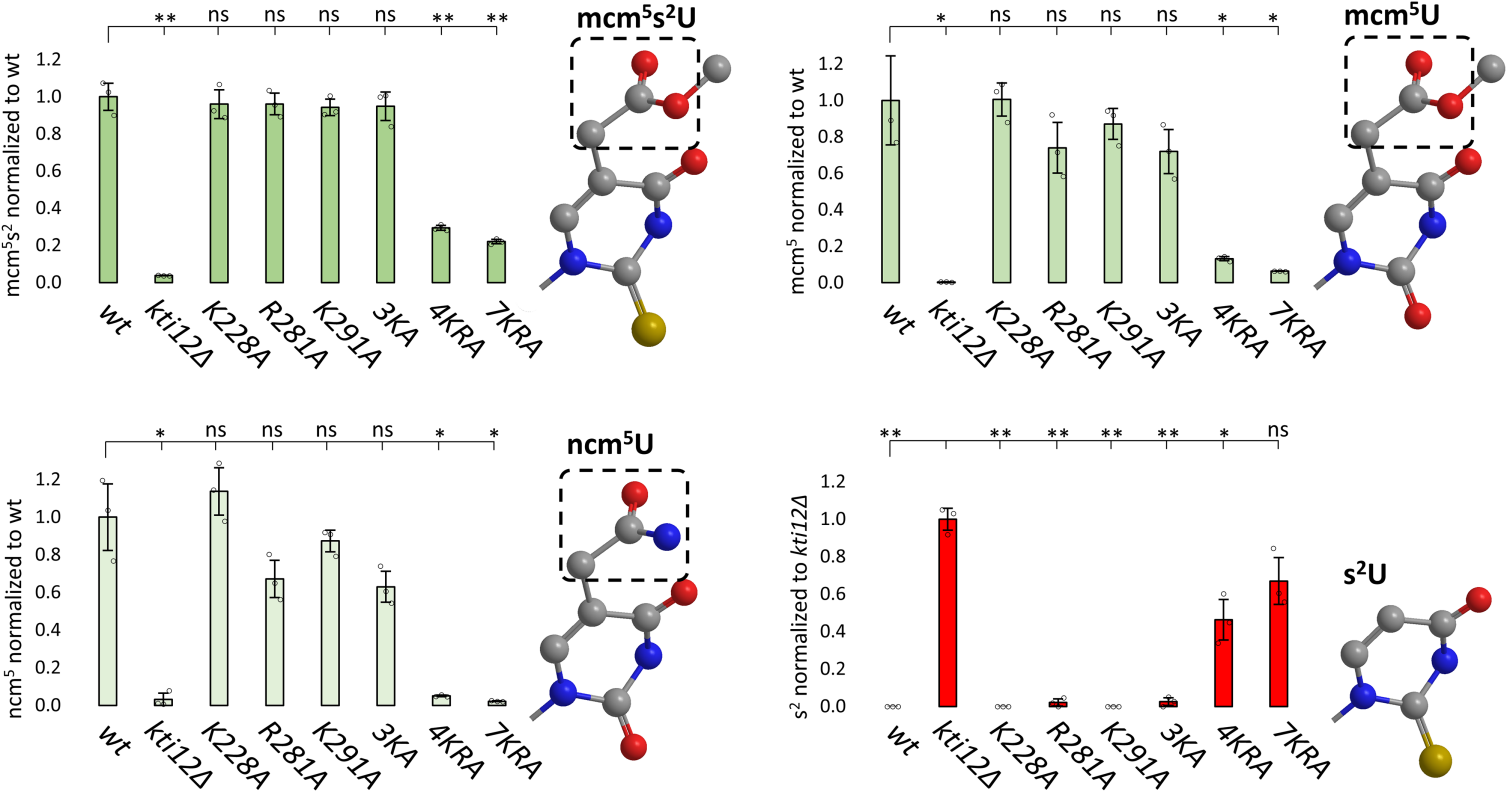
The CTD in Kti12 promotes wobble uridine modification by Elongator. LC-MS/MS quantification of Elongator dependent mcm^5^s^2^U, mcm^5^U and ncm^5^U modifications (different shades of green) from the indicated *kti12* mutants and controls (*kti12*Δ, wt). The initial carboxymethylation reaction by Elongator is indicated by the dashed box. Detection of s^2^U is a telltale sign of Elongator inactivity (hence labeled by red bars) since the thiolation occurs independently of the C5 modification [58–60]. Measurements were performed from biological triplicates. Error bars indicate standard deviation, and statistical significance was tested using a two-tailed t-test (** *P* < 0.01, * *P* < 0.05, ns: not significant).

To examine the role that the tRNA binding CTD of Kti12 plays for Elongator function *in vivo*, we used previously described mass spectrometry (LC-MS/MS) protocols [38, 41, 58, 59] and compared relative abundances of U_34_ modifications between a *KTI12* wild-type strain, a *kti12*Δ null-mutant and different CTD mutants (i.e. K228A, R281A, K291A, 3KA, 4KRA, and 7KRA) (Fig. 4). We found that in contrast to single mutations (i.e. K228A, R281A, and K291A), the combination of CTD substitutions in helix α9 (4KRA) or α9/α10 (7KRA) drastically reduced Elongator dependent formation of ncm^5^U_34_, mcm^5^U_34_ and mcm^5^s^2^U_34_ derivatives (Fig. 4). With defects in mcm^5^U_34_ and mcm^5^s^2^U_34_ modification types previously shown to block tRNA suppressor *SUP4* and deny tRNA cleavage by γ-toxin, respectively [5, 45, 56], our LC-MS/MS profiles are in line with the above *SUP4* and γ-toxin assays (Figs 1 and 2) that diagnosed Elongator dysfunction in CTD mutants (4KRA, 7KRA). Moreover, as a result of Elongator defects that likely associate with Kti12 inactivation, our LC-MS/MS profiles reveal elevated amounts of s^2^ at U_34_ in the affected CTD mutants (i.e. 4KRA, 7KRA) also found in *kti12*Δ cells (Fig. 4). s^2^U_34_ is a thio-modification usually not detected in endogenous tRNAs from Elongator wild-type yeast cells, which only appears when U_34_ cannot be properly modified to mcm^5^s^2^U_34_ by Elongator (e.g *elp3*Δ) [58–60]. Thus, elevated s^2^U_34_ levels further indicate Elongator dysfunction in the CTD mutants (4KRA and 7KRA). In summary, altered LC-MS/MS profiles (Fig. 4) support our view above that *kti12* mutants, which lack tRNA binding due to multiple CTD substitutions (4KRA and 7KRA), copy *kti12*Δ loss-of-function scenarios and suppress Elongator dependent U_34_ modifications *in vivo*.

### The CTD of Kti12 is also important for Elongator interaction

ATP binding in the NTD and ATP hydrolysis by Kti12 are known to be required for Elongator function [41]. Here we demonstrate that substitutions in the CTD that affect tRNA binding have a similar effect (Fig. 3). This strongly suggests that the integrity of both domains is crucial for Kti12 function and Elongator activity. In addition, we show that the expression of each domain alone neither supported Elongator function (Supplementary Fig. S3A) nor was it able to mediate proper protein–protein interaction seen between full-length Kti12 and Elongator *in vivo* (Supplementary Fig. S3B). Next, we examined potential effects of CTD mutations on the interaction between Elp1-c-Myc and Kti12-HA *in vivo* using Co-IP.

**Figure 5.**
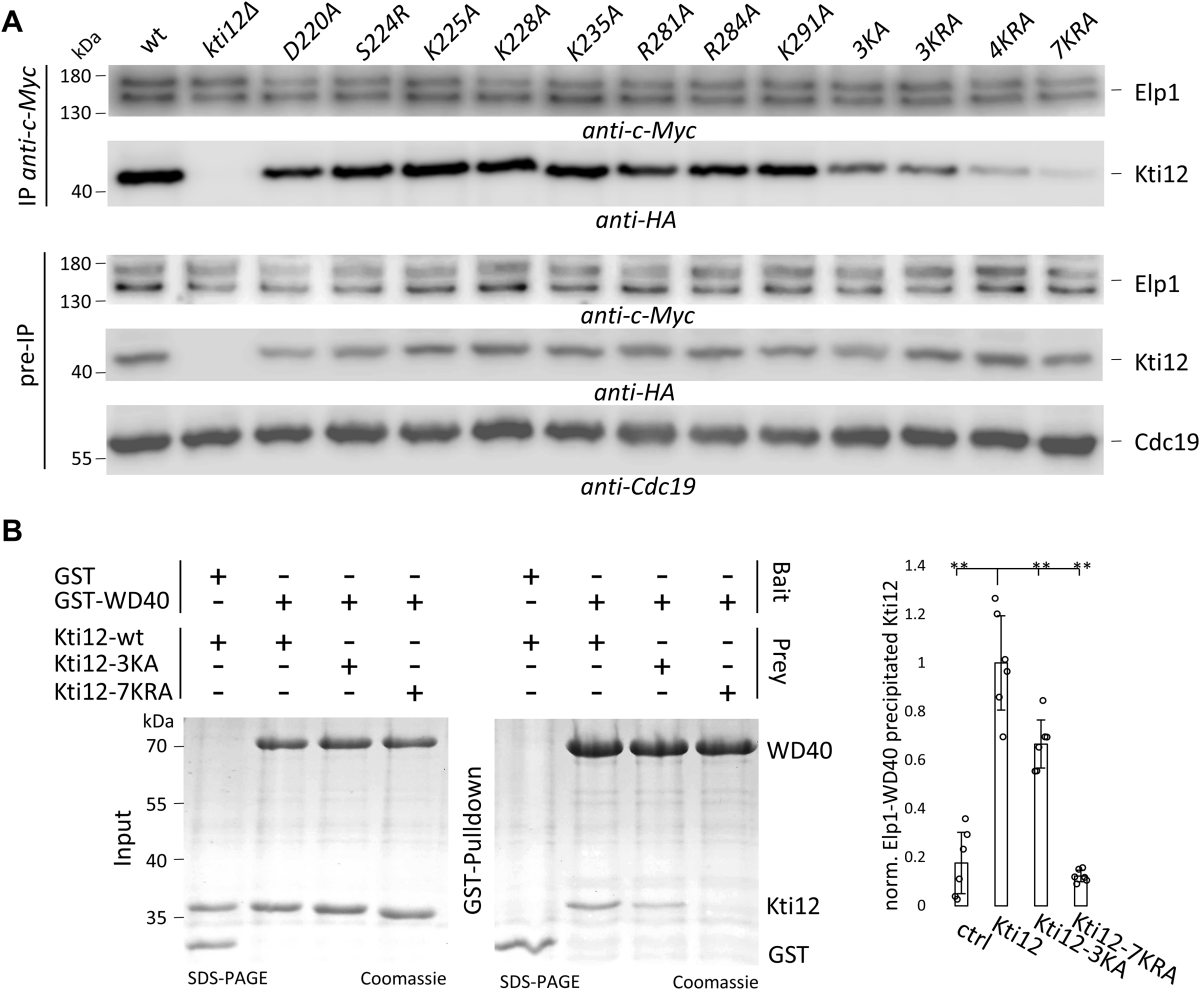
The Kti12 CTD is important for Elongator interaction. **(A)** Co-IP of Elp1 and Kti12 from an *ELP1-c-myc* and *KTI12-HA* tagged strain. Elp1-c-Myc was immobilized and Kti12-HA precipitation was detected via Western blots. The anti-Cdc19 antibody was used to control protein loading. **(B)** GSTpull-down (left panel) of recombinant Kti12 mutants and the NTD of Elp1 (WD40 aa1-734, see Supplementary Fig. S4, for details). Cumulative CTD substitutions attenuate direct Kti12 interaction with Elp1. Interaction quantifications (right panel) were performed in triplicates. Error bars indicate standard deviation and statistical significance was tested using a two-tailed t-test (** *P* < 0.01, * *P* < 0.05).

As judged from these experiments, all CTD substitutions were expressed *in vivo*. None of the single CTD substitutions had a discernible effect on Kti12 interaction with Elp1 (Fig. 5A). However, the combined CTD mutations showed progressive loss of Elongator interaction that correlates with the number of substituted residues (3KA < 3KRA < 4KRA < 7KRA) and the severity of their tRNA binding deficiencies (Fig. 3), suggesting that the residues in Kti12 needed for tRNA binding are also necessary for the physical contact with Elongator (Fig. 5A). Since Elp1 has been shown to bind Kti12 *in vitro* via WD40 motifs in its own NTD [41], we performed direct GST pull-down experiments between the first WD40 domain of Elp1 (aa42-431: Supplementary Fig. S4) and wild-type Kti12 or CTD mutants (3KA, 7KRA). As shown from these interaction assays, the direct binding between Elp1 and Kti12 depends on an integer CTD region *in vitro* and the CTD mutants (3KA, 7KRA) gradually lose the ability to get captured by the GST-tagged Elp1 bait (Fig. 5B). Thus, additive alanine substitutions in helices α9/α10 of the CTD likely lead to a cumulative decline in the ability of Kti12 to associate with Elongator *in vivo* (Fig. 5A) or the Elp1 subunit *in vitro* (Fig. 5B). In sum, this body of evidence complements our genetic and biochemical data above further supporting the notion that the CTD capable to bind tRNA also supports Kti12 interaction with Elp1. A direct pull-down of Kti12 by Elp1 in the absence of tRNA (Supplementary Fig. S4) shows that tRNA is not required for the contact between Kti12 and Elp1 *in vitro*.

### tRNA binding *in vivo* distinguishes free from Elongator bound pools of Kti12

**Figure 6.**
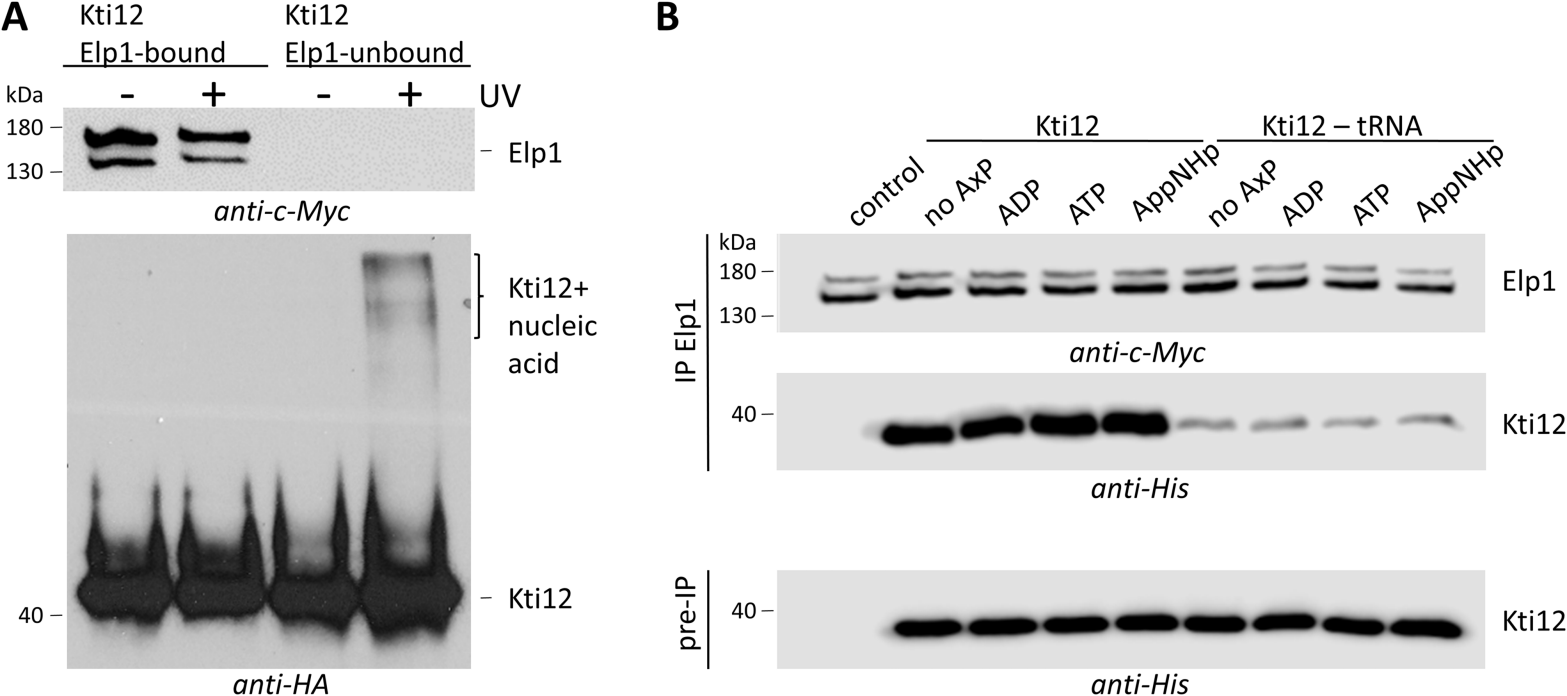
Kti12 mainly binds tRNA separate from Elongator. **(A)** Protein extracted from *ELP1-c-mycKTI12-HA* expressing cells was subjected to anti-c-Myc IP to precipitate Elp1. From these Elongator immune depleted fractions, Kti12-HA was enriched by a second anti-HA IP and irradiated with (+) UV (254 nm) to induce nucleic acid cross-links or without UV (-). Subsequently, immune blots of both IP samples were probed with anti-c-Myc and anti-HA antibodies. Note that UV treatment shifts the electrophoretic mobility of Elp1-unbound Kti12 material to a higher molecular weight complex likely composed of a cross-linked nucleic acid. This is in marked contrast to the behavior of Elp1-bound Kti12 pools. **(B)** Elongator was precipitated from a *kti12*Δ strain expressing Elp1-c-myc by IP (see A) and served for association assays, in which recombinant His-tagged Kti12 material preincubated without (no AxP) or with nucleotides (i.e. ATP, ADP, or non-hydrolysable AppNHp) was used in the absence (left panel half: Kti12) or the presence of bulk tRNA (right panel half: Kti12-tRNA).

Prompted by previous data showing that there are Elongator bound and unbound pools of Kti12 [17, 33, 61], we decided to separately examine the potential tRNA binding capacity of these different forms. Therefore, total protein was extracted from a yeast strain (*ELP1-c-myc*, *KTI12-HA*) that co-expresses c-Myc- and HA-tagged forms of Elp1 and Kti12, and subjected to IP using anti-c-Myc antibodies. The resultant Elongator immune depleted fraction was further precipitated with anti-HA antibodies to enrich for the free form of Kti12 that is not bound to Elongator. Next, SDS-PAGE and Western blots were carried-out with anti-c-Myc and anti-HA antibodies to analyse the content of Elp1-c-Myc and/or Kti12-HA in each IP sample. The data show that free forms of Kti12 can be enriched upon Elongator depletion and hence separated from Elongator-bound pools of Kti12 (Fig. 6A).

To further study whether these Kti12 fractions may differ in tRNA binding *in vivo*, we resorted to a UV cross-linking IP (UV-CLIP) technique previously shown to trap more transient nucleic acid–protein complexes [52, 62]. We split our IP samples, irradiated half of the Elongator bound and unbound Kti12 pools with UV_254nm_ light to induce cross-linking between protein and nucleic acids. Next, untreated and irradiated material were subjected to Western Blot, and cross-linked species with retarded mobility were detected only in the free, Elongator-unbound Kti12 fraction (Fig. 6A). Thus, only free Kti12 is able to form a complex with nucleic acids *in vivo*, confirming the results obtained with recombinant Kti12, which is able to bind tRNA *in vitro* (Fig. 3). Such scenario, in which tRNA is differently recognized by Elongator-bound and unbound Kti12 pools (Fig. 6A), suggests that Kti12 may act as a tRNA deposition module for Elongator. Since ATP binding and hydrolysis by Kti12 are also critical for Elongator function [41], we analyzed whether the binding to either Elongator or tRNA may change in response to nucleotide treatment. Hence, we enriched Elongator from a *kti12*Δ mutant expressing c-Myc tagged Elp1 by anti-c-Myc IP and combined the precipitates with nucleotide-preincubated Kti12 material in absence or presence of tRNA (Fig. 6B). Robust association between recombinant Kti12 and immune purified Elongator was detectable in the absence of tRNA (Fig. 6B) and in the presence of any of nucleotides (i.e. ATP, ADP, or non-hydrolysable AppNHp) tested. Kti12 samples pre-incubated with tRNA, however, showed drastically reduced interaction levels with Elongator. Even though the interaction occurred irrespective of whether or not the samples were treated with nucleotides (Fig. 6B), the data correspond with the UV-CLIP assays above (Fig. 6A) as they demonstrate that Kti12 mainly binds tRNA in a fashion separate from Elongator.

Although several options are possible to explain this tRNA specific effect, one possible scenario may include the competition for tRNA binding between Kti12 and Elongator. Therefore, we determined the tRNA affinity of Kti12 using different tRNA concentrations (Supplementary Fig. S5). We observed K_D_ values ranging from ∼1.3–1.6 μM (Supplementary Fig. S5), suggesting a tRNA affinity for Kti12 that is weaker than the fully assembled Elongator complex (∼200 nM), the Elp123 subcomplex (∼70 nM) or the Elp3 subunit alone (∼600 nM) [1, 39, 62, 63]. Whether the weaker interaction implies a role for Kti12 as a tRNA carrier that assists the Elongator complex by acting as a tRNA deposition factor rather than a catalyst in the U_34_ modification pathway is an intriguing option that needs to be further elucidated in the future.

## Conclusions

Although PSTK and Kti12 are structurally very similar, they differ in function. While PSTK is part of tRNA^Sec^ synthesis for UGA translational recoding and selenocysteine incorporation into proteins [40, 64], Kti12 appears to act as a tRNA delivery factor rather than a catalyst for Elongator in the U_34_ modification pathway [37, 41]. Our data confirm that similar to PSTK, Kti12 binding to tRNA is mediated by multiple positively charged amino residues in the CTD, i.e. helix α9 and helix α10 (Fig. 1) [40, 65]. As a consequence, cumulative CTD substitutions (4KRA and 7KRA) disrupt the interaction with tRNA, inactivate Kti12 function and essentially, suppress the U_34_ modification activity of Elongator *in vivo*. These negative effects on Elongator activity are comparable to ATPase mutations in the NTD (K14A; D85A), which previously indicated that ATP hydrolysis by Kti12 is required for Elongator function, too [41]. Thus, binding to both, ATP and tRNA, drives the function of Kti12 and ultimately, the U_34_ modification activity of the Elongator complex. Therefore, more research is needed to clarify the ill-defined role of the ATPase activity [41] and to address how ATP hydrolysis and tRNA binding are coupled in Kti12 to support Elongator's tRNA modification activity. Whether and how Kti12 helps to provide Elongator with substrate tRNAs is important to understand mechanisms and conditions underlying Elongator regulation. Here, comparison of dissociation constants for tRNA in complex with either Elongator or Kti12 may be helpful and clarify the precise roles that Elongator bound and unbound Kti12 pools play in the U_34_ modification pathway.

In summary, our study provides further evidence that the U_34_ modification pathway depends on a tRNA carrier protein dedicated to Elongator, namely Kti12. It seems likely that Kti12 together with other regulatory factors (i.e. Kti11, Kti13, and Kti14) forms part of a dynamic protein network [27], whose associations with the Elongator complex appear to be sensitive to certain metabolic signals (i.e. nucleotides; SAM; acetyl-CoA) and substrate tRNA [4, 41]. In support of this notion it was shown that loss of U_34_ modifications in Elongator mutants confers altered metabolic profiles [66] and sensitivity to various stress conditions including inhibition of the TOR pathway [67, 68], a central growth controller that coordinates nutritional signals with cell proliferation. Thus, tRNA modifications dependent on Kti12 and Elongator appear to provide cells with means that amongst other epitranscriptomic marks, can contribute to tRNA modification biased gene expression and stress-specific cell responses [28–30, 69, 70].

## Supplementary Material

gkaf296_Supplemental_File

## Data Availability

All data are incorporated into the article and its online Supplementary Material.
