# Supplementary material for: tRNA binding to Kti12 is crucial for wobble uridine modification by Elongator
Source: Nucleic Acids Res. 2025 Apr 14;53(7):gkaf296. doi: 10.1093/nar/gkaf296 (PMC11995267; doi:10.1093/nar/gkaf296)
Supplement: gkaf296_Supplemental_File [file gkaf296_supplemental_file.pdf]

Supplementary Material

tRNA binding to Kti12 is crucial for wobble uridine modification by Elongator

David Scherf[1], Alexander Hammermeister[1,2], Pauline Böhnert[1], Alicia Burkard[3], Mark Helm[3], Sebastian Glatt[2,4] and Raffael Schaffrath[1]*

[1] Institute of Biology, Division of Microbiology, University of Kassel, D-34132 Kassel, Germany

[2] Małopolska Centre of Biotechnology, Jagiellonian University, 30387, Krakow, Poland

[3] Institute of Pharmaceutical and Biomedical Sciences, Johannes Gutenberg University of Mainz, D-55128 Mainz, Germany

[4] Department for Biological Sciences and Pathobiology, University of Veterinary Medicine Vienna, Vienna, Austria

* To whom correspondence should be addressed. Email: schaffrath@uni-kassel.de; Tel: +49 561 804-4175.

1. Supplementary Tables

Table S1. Yeast strains used and generated in this study.

Strain	Genotype	Reference
Saccharomyces cerevisiae:		
UMY2893	*MATα SUP4 leu2-3,112 trp1-1 can1-100 ura3-1 ade2-1 his3-11,15*	(1)
UMY2916	Like UMY2893, but *Δelp3::KanMX4*	(1)
yAH108	Like UMY2893, but *ELP1-(c-myc)$_3$::SpHIS5, KTI12-HA$_6$::KlTRP1*	This study
yAH102	Like yAH108, but *Δkti12::loxP*	This study
ySF11	Like yAH102, but *kti12-D220A-HA$_6$::KlTRP1*	This study
ySF12	Like yAH102, but *kti12-S224R-HA$_6$::KlTRP1*	This study
ySF17	Like yAH102, but *kti12-K225A-HA$_6$::KlTRP1*	This study
ySF18	Like yAH102, but *kti12-K228A-HA$_6$::KlTRP1*	This study
ySF19	Like yAH102, but *kti12-K235A-HA$_6$::KlTRP1*	This study
ySF13	Like yAH102, but *kti12-R281A-HA$_6$::KlTRP1*	This study
ySF328	Like yAH102, but *kti12-K283A-HA$_6$::KlTRP1*	This study
ySF20	Like yAH102, but *kti12-R284A-HA$_6$::KlTRP1*	This study
ySF21	Like yAH102, but *kti12-K291A-HA$_6$::KlTRP1*	This study
ySF15	Like yAH102, but *kti12-K225A, K228A, K235A-HA$_6$::KlTRP1*	This study
ySF22	Like yAH102, but *kti12-R281A, K283A, R284A-HA$_6$::KlTRP1*	This study
ySF16	Like yAH102, but *kti12-R281A, K283A, R284A, K291A-HA$_6$::KlTRP1*	This study

ySF23	Like yAH102, but *kti12-K225A, K228A, K235A, R281A, K283A, R284A, K291A-HA*$_6$*::KlTRP1*	This study
yAH76	Like UMY2893, but *ELP1-(c-myc)*$_3$*::SpHIS5, Δkti12::KlURA3*	(2)
RZY144	Like yAH76, but *kti12-K14A-HA*$_6$*::KlTRP1*	(2)
RZY150	Like yAH76, but *kti12-D85A-HA*$_6$*::KlTRP1*	(2)
Kluyveromyces lactis:		
AWJ137	*MATa leu2 trp1* [k1$^+$/k2$^+$]	(3)

Table S2. Primers used in this study.

Primer name	Usage	Sequence 5′- 3′
Fragment_Kti12_Fwd	Insertion	gtcatcgtcatcgcgatgga
Kti12_Rev		gatacccagttgagaagacgagc
Kti12_uORF_exchange_Fw		ctcataccaaccggaaagga
Kti12_ko_Rv	Gene knock-out	agcaaatttcgtcttgccatttaccttctgatattaatcacatgtatatcgcataggccactagtggatctg
Kti12_ko_Fw		gcatatttatataaggaaatattagttgcaaatcattatgtcattcctttccagctgaagcttcgtacgc
Kti12_D220A_Fw	Site-directed mutagenesis	catccaggttctcgccatcgaaac
Kti12_D220A_Rv		ctagtttcgatggcgagaacctggatg
Kti12_S224R_Fw		gacatcgaaactcgtaagataataaaaacc
Kti12_S224R_Rv		ggtttttattatcttacgagtttcgatg
Kti12_K225A_Fw		cgaaactagtgcgataataaaaac
Kti12_K225A_Rev		ggtttttattatcgcactagtttc
Kti12_K228A_Fw		ctagtaagataatagcaaccataatgaacc
Kti12_K228A_Rv		tctattatcgttggtattacttggtgtag
Kti12_K235A_Fw		ccacatcgcaagcctgac
Kti12_K235A_Rv		gaagtcaggcttgcgatgtg
Kti12_R281A_Fw		gcaattgcaggcattgaaaaggcaatt
Kti12_R281A_Rv		ccgcgttaacgtccgtaacttttc
Kti12_K283A_Fw		cagagattggcaaggcaa
Kti12_K283A_Rv		gaattgccttgccaatctct
Kti12_R284A_Fw		gagattgaaagcgcaattcattaactt
Kti12_R284A_Rv		taatgaattgcgctttcaatctctgcaatt
Kti12_K291A_Fw		cattaacttcaacgcactaagagatatag
Kti12_K291A_Rv		atctcttagtgcgttgaagttaatgaattgc
Kti12_K225-K228-K235-A_Fw		cgacatcgaaactagtgcgataatagcaaccataatgaaccacatcgcaagcctgacttct
Kti12_K225-K228-K235-A_Rv		ccaatagaagtcaggcttgcgatgtggttcattatggttgctattatcgcactagtttcgat
Kti12_R281-K283-R284-A_Fw		gcgcaattgcaggcattggcagcgcaattcatt
Kti12_R281-K283-R284-A_Rv		gttaatgaattgcgctgccaatgcctgcaattgcg
Kti12_R281-K283-R284-K291-A_Fw		gcaattgcaggcattggcagcgcaattcattaacttcaacgcactaagag
Kti12_R281-K283-R284-K291-A_Rev		ctatatctcttagtgcgttgaagttaatgaattgcgctgccaatgcctgca

2. Supplementary Figures

Figure S1:

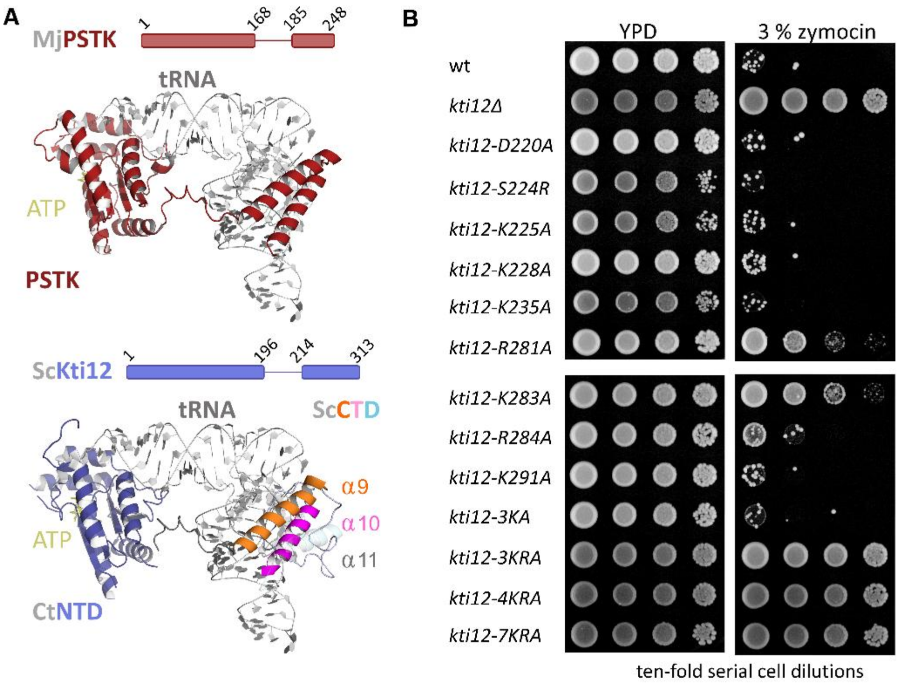

Figure S1. The PSTK like protein Kti12 and its function for Elongator. (A) Model comparison of PSTK (PDB 3adb) and Kti12. (B) Growth analysis of indicated Kti12 CTD substitution mutants (for residue details, see Fig. 1) in response to exogenous zymocin, an Elongator dependent tRNase killer toxin from dairy yeast *K. lactis*. The *KTI12* wild-type (wt) and null mutant (*kti12*Δ) served as zymocin sensitive and resistant controls, respectively.

Figure S2:

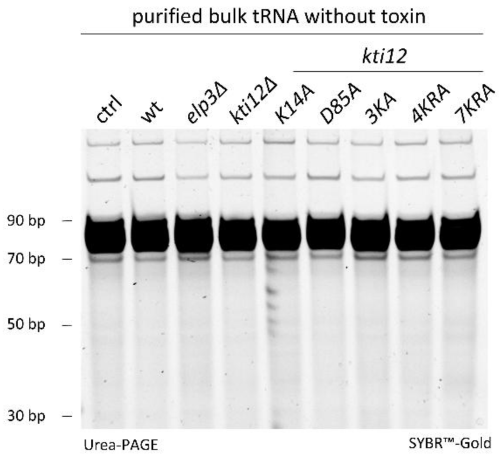

Figure S2. Untreated purified bulk tRNA from the indicated genetic strain backgrounds. The samples were monitored by UREA-PAGE prior to treatment and cleavage by purified γ-toxin tRNase.

Figure S3:

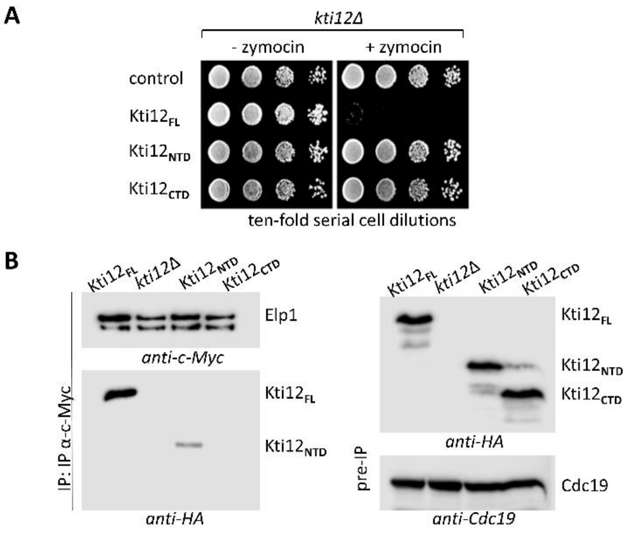

Figure S3. Both Kti12 domains are required for Elongator function. (A) Vector based expression of full-length Kti12, the Kti12$_{NTD}$ (aa1-182) or Kti12$_{CTD}$ (aa183-313) under native promoter in a *kti12Δ* background (control = empty vector). (B) Elp1 Co-IP from the *kti12Δ* background (A) indicates that neither Kti12$_{NTD}$ nor Kti12$_{CTD}$ interact with Elp1 *in vivo* comparable to Kti12$_{FL}$.

Figure S4:

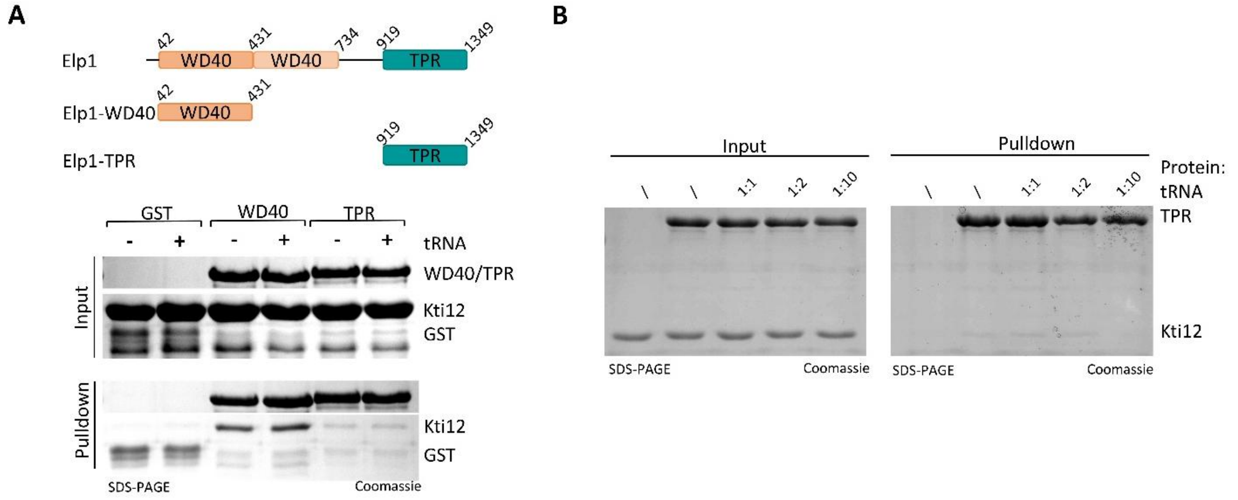

Figure S4. tRNA does not attract Kti12 to Elp1 domains *in vitro*. (A) The N-terminal WD40 (aa42-431) domain and C-terminal TPR domain (aa919-1349) of Elp1 were GST tagged and used as a bait for Kti12. The experiment was carried out in the absence and presence of purified tRNA (Molar ratio 1:10:2.5 Elp1:Kti12:tRNA). (B) Similar to (A), the interaction of Elp1-TPR and Kti12 was analyzed with rising amounts of tRNA (Molar ratio 1:1:0.01-5 TPR:Kti12:tRNA).

Figure S5:

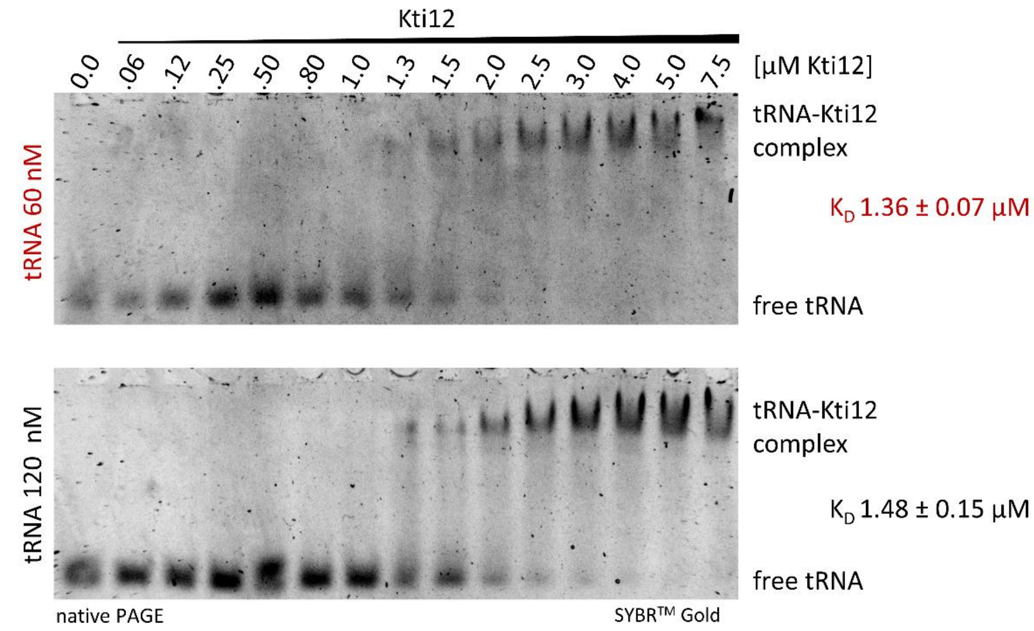

Figure S5. tRNA affinity of Kti12 from yeast. K_D determinations involved recombinant Kti12 with bulk tRNA (0.06 & 0.12 µM) prepared from wild-type yeast. Shown are electrophoretic mobility shift assays (EMSA) stained with SYBR gold to detect tRNA that is either free or in complex with Kti12. Estimated K_D values represent standard deviations from three experiments.

3. Supplementary References

1. Huang, B., Johansson, M.J.O. and Byström, A.S. (2005) An early step in wobble uridine tRNA modification requires the Elongator complex, *RNA (New York, N.Y.)*, **11**, 424–436.
2. Krutyhołowa, R., Hammermeister, A., Zabel, R., Abdel-Fattah, W., Reinhardt-Tews, A., Helm, M., Stark, M.J.R., Breunig, K.D., Schaffrath, R. and Glatt, S. (2019) Kti12, a PSTK-like tRNA dependent ATPase essential for tRNA modification by Elongator, *Nucleic Acids Research*, **47,** 4814–4830.
3. Kämper, J., Esser, K., Gunge, N. and Meinhardt, F. (1991) Heterologous gene expression on the linear DNA killer plasmid from *Kluyveromyces lactis*, *Curr Genet,* **19**, 109–118.
